# Assessing the Implementation and Potential Effects of the Nishauri mHealth Intervention on HIV Care Among Men in Homa Bay County, Kenya: Protocol for a Mixed Methods Study

**DOI:** 10.2196/85279

**Published:** 2026-03-24

**Authors:** Dan Omollo, Pamela Murnane, Louisa Ndunyu, Dallas Swendeman, Sheri D Weiser, Collins Ouma

**Affiliations:** 1 Department of Epidemiology and Biostatistics, Global Health Institute, University of California, San Francisco, 590 Minnesota, San Fransisco, CA, 94107, United States, 1 6282463634; 2 Department of Public Health, School of Public Health and Community Development, Maseno University, Kisumu, Kenya; 3 Department of Epidemiology and Biostatistics, University of California, San Francisco, San Francisco, CA, United States; 4 Semel Institute for Neuroscience and Human Behavior, University of California, Los Angeles, Los Angeles, CA, United States; 5 Department of Medicine, University of California, San Francisco, San Fransisco, CA, United States; 6 School of Public Health and Community Development, Maseno University, Kisumu, Kenya

**Keywords:** mHealth, mobile health, HIV care, men living with HIV, implementation, masculinity, Kenya, Nishauri

## Abstract

**Background:**

About 2.4% of Kenyan people (approximately 1.3 million people) are living with HIV. Despite advances in antiretroviral therapy, men continue to experience disproportionately poor engagement in HIV care due to entrenched masculine norms, stigma, and lack of tailored interventions. Mobile health (mHealth) platforms offer a promising strategy to improve care engagement, but evidence on its implementation and impact among men living with HIV is limited.

**Objective:**

This study aims to assess the implementation and potential effects of the Nishauri mHealth intervention on HIV care and treatment outcomes among men in Western Kenya. Specifically, it seeks to (1) analyze its effects on HIV care engagement and treatment outcomes, (2) explore the role of masculine identity in modifying acceptability and uptake, and (3) identify barriers and facilitators of adoption, use, and sustainment.

**Methods:**

We will use a mixed methods design combining a stepped-wedge cluster approach and a pre- and postimplementation assessment across 4 health facilities in Homa Bay County, Kenya. Approximately 347 men receiving HIV treatment who own a smartphone will be enrolled. The stepped-wedge design will sequentially introduce the intervention across the 4 facilities in 2-month intervals following baseline assessments, allowing each site to serve as its own control. Surveys will collect data on sociodemographics, masculinity, intervention acceptability and uptake, and HIV clinical outcomes using validated measures. Intervention effects on pre- and postbinary outcomes will be assessed using the McNemar test, while generalized estimating equations (*α*=.05; *β*=.2; 95% CI) will account for clustering and repeated measures in the stepped-wedge analysis. Focus group discussions (n=5-6) will be conducted with men living with HIV, health care providers, and app developers to explore barriers and facilitators of implementation and adoption. Focus group discussions will be audio-recorded, transcribed, coded, and analyzed thematically.

**Results:**

This study received institutional review board approval in July 2025 and was registered on ClinicalTrials.gov in August 2025. Recruitment began in September 2025 and concluded in November 2025. A total of 307 men living with HIV were recruited across the 4 clinics for the pre- and postquantitative assessment. Preliminary findings will describe implementation outcomes and early effects on HIV care engagement.

**Conclusions:**

This trial will use a stepped-wedge design to evaluate the implementation and effects of the Nishauri mHealth intervention on antiretroviral therapy adherence and clinic attendance among men in Homa Bay County. By examining both clinical outcomes and the influence of masculine norms on intervention uptake, it will provide robust evidence on the effectiveness of mHealth strategies tailored for men in low-resource, high–HIV-burden settings. Findings will inform the design, scalability, and optimization of similar interventions by identifying key implementation barriers and facilitators.

## Introduction

Globally, men are 27% less likely than women to seek HIV testing and often present to care at later stages of illness [1]. In sub-Saharan Africa, men’s participation in HIV care is further constrained by cultural expectations that prioritize economic provision, requiring men to focus on work and income generation over health-seeking. Additionally, norms around emotional suppression discourage men from expressing vulnerability or seeking support, including attending health services [2,3]. Recent studies highlight that men who conform to traditional masculine norms are less likely to attend routine HIV care and more likely to disengage from treatment [4-6]. Despite advances in antiretroviral therapy (ART) and HIV care services, men experience persistent barriers to accessing and engaging with care. Masculine norms around strength, stoicism, and self-reliance often discourage men from seeking health services, including HIV testing and treatment [4], contributing to lower engagement and adherence [7,8]. The lack of men-targeted interventions compounds these challenges, making it critical to develop and evaluate strategies that effectively engage men in care to achieve the Joint United Nations Programme on HIV/AIDS 95-95-95 targets by 2030.

Mobile health (mHealth) interventions have emerged as promising tools for improving health care delivery and patient engagement, particularly in resource-constrained settings [8-10]. By leveraging mobile devices to deliver health-related information and services, mHealth strategies help bridge critical gaps in access, communication, and continuity of care. Evidence from low- and middle-income countries indicates that mHealth interventions, such as SMS reminders and mobile apps, can improve ART adherence, retention in care, and health literacy [4,7] among people living with HIV. Studies globally and in sub-Saharan Africa have shown the potential of mHealth interventions to enhance clinical outcomes, reduce loss to follow-up, and strengthen engagement in care [4,7,10-13]. For instance, studies in Uganda and South Africa show that mobile reminders and SMS-based counseling significantly improve adherence and retention among individuals with HIV [14-16]. These tools facilitate behavior change through personalized messaging, medication reminders, and educational content. Critically, culturally adapted mHealth interventions targeted for men are more effective, as they address stigma and align health-seeking behaviors with socially accepted masculine norms. For example, SMS reminders framed around men’s responsibilities or apps providing discreet, personalized health information have been shown to improve ART adherence and clinic attendance among men in sub-Saharan Africa [14-17]. This demonstrates how aligning messages and delivery with socially accepted masculine norms can reduce stigma and enhance engagement. However, there remains limited evidence on the implementation outcomes, service outcomes, and long-term sustainability of mHealth interventions specifically tailored for men in HIV care in Kenya, a country that continues to face significant challenges in the fight against HIV/AIDS, with an estimated 2.4% of the population being HIV-seropositive [18].

In Kenya, the Nishauri (client-centered mHealth digital) platform was developed to enhance HIV care management by supporting appointment reminders, ART adherence, patient-provider communication, and educational messaging. Pilot implementations of Nishauri and its predecessor, Ushauri, have shown promise in improving engagement and retention, though systematic evidence on its adoption and sustained use, particularly among men, remains limited [19,20]. While mHealth technologies such as Nishauri have shown promise in improving HIV care outcomes [21,22], their adoption and sustained use in Kenya remain at an early stage and face significant challenges. Structural barriers, including digital literacy gaps, inconsistent device ownership, limited infrastructure, and concerns around data privacy and confidentiality, continue to hinder equitable uptake [23,24]. Mobile phone ownership and digital access also vary by gender, education level, and socioeconomic status [25], with men in rural, high-prevalence settings like Homa Bay County often facing compounded disadvantages. Beyond structural issues, many mHealth interventions are not adequately aligned with the gendered and sociocultural realities that shape care-seeking behaviors. Programs often lack meaningful provider involvement and fail to tailor delivery to address male-specific barriers or local norms [26]. Although designed to be discreet and convenient, mHealth tools may fall short if they do not resonate with prevailing masculine identities or reframe care-seeking in ways that affirm strength and responsibility [17]. In high-burden regions such as Homa Bay, where HIV prevalence is 16.2% [18,27], men frequently disengage from care due to stigma and perceived threats to masculinity [28].

In parts of Homa Bay and other counties where Nishauri has been implemented on a pilot basis for over 6 months, its uptake and use remain low and highly variable across facilities, providers, and patient populations. This variability hints at challenges with its implementation and effectiveness among men, a population that faces unique sociocultural barriers to HIV care. Existing research often overlooks the role of gender norms and contextual factors in influencing men’s engagement with digital health interventions. Addressing this gap is essential for designing scalable, culturally relevant mHealth solutions that promote sustained engagement and improved clinical outcomes among men. This study will be guided by an integrated conceptual framework combining Connell’s theory of hegemonic masculinity [29] and the technology acceptance model (TAM) [30,31] to underpin the design and evaluation of Nishauri, predicting how individual factors (eg, perceived usefulness, usability, and masculinity norms) and system-level factors (eg, workflow integration and provider support) influence adoption, engagement, and sustainment of the intervention.

This study aims to assess the implementation and potential effects of the Nishauri mHealth intervention on HIV care and treatment outcomes among men aged 18‐55 years in Homa Bay County, Western Kenya. Specifically, it seeks to (1) analyze its potential effects on HIV care and treatment outcomes, (2) explore the role of masculine identity in modifying its acceptability and uptake, and (3) identify barriers and facilitators of its adoption, use, and sustainment.

## Methods

### Study Design

**Figure 1.**
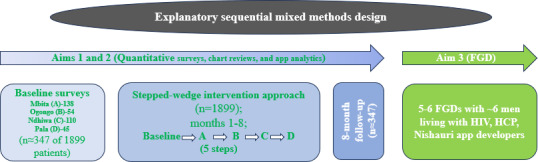
Study Schema. FGD: focus group discussion; HCP: healthcare providers.

This study will use an explanatory sequential mixed methods design, combining quantitative and qualitative approaches to understand both implementation outcomes and contextual factors influencing intervention uptake [32]. The quantitative component will use a stepped-wedge approach across 4 health facilities (n=1899) [33,34] analyzed using generalized linear mixed models to account for clustering and repeated measures [34,35] with pre- and postassessment of implementation outcomes [36,37] (n=347; Figure 1). The qualitative component will involve thematic analysis of focus group discussions (FGDs; 5‐6 groups of ~6 key stakeholders) across 4 health facilities in Homa Bay County (Table 1). This analysis will combine deductive coding guided by Proctor’s implementation outcomes framework with inductive coding to capture emergent themes [36]. This integrated approach ensures rigorous evaluation of the Nishauri mHealth intervention across multiple levels and stakeholder perspectives.

**Table 1.** Focus group discussion (FGD) sampling plan or rationale.

Group	FGDs, n	Participants per FGD, n	Total participants, n	Plan or rationale
Men living with HIV	2‐3	6‐8	12‐24	Stratified by age, missed visits, antiretroviral therapy adherence, and intervention adoption.
Health care providers	2	6‐8	12‐16	Grouped by facility or role (eg, nurse vs adherence counselor). County or subcounty officials to provide valuable insight into policy, scale-up, and sustainability.
App developers	1	4‐6	4‐6	Being a homogeneous group, 1 FGD session should suffice.

After baseline data collection is completed, a standardized readiness assessment will be conducted across the 4 facilities to determine the procedure of the phased intervention rollout. We will leverage a partnership with the Palladium Group (the app developers) to access backend use data from the Nishauri app, providing detailed insights into how different app functions are used in real-world settings. This mixed methods design will enable both within- and between-facility comparisons, offering a nuanced understanding of how implementation processes and gendered experiences shape the intervention’s effectiveness among men. This study protocol was developed and reported in accordance with the SPIRIT (Standard Protocol Items: Recommendations for Interventional Trials) 2013 guidelines (Checklist 1).

### Study Setting

We will conduct this study in 4 subcounty HIV Comprehensive Care Clinics (CCC) in Homa Bay County, Kenya—Mbita, Ogongo, Ndhiwa, and Pala. Homa Bay County was chosen for its highest HIV prevalence, 16.2% [18] in Kenya, making it suitable to assess the implementation process and outcomes of an mHealth intervention for enhancing HIV care. The county covers an area of about 3154 km^2^ [38,39]. As of the 2019 Kenya National Census, it had an estimated population of approximately 1.1 million people [39]. There are approximately 160 health facilities providing HIV comprehensive care services across the county. ART uptake is at 96%, while viral load suppression (VLS) prevalence is at 83.8% among people living with HIV, with significant disparities between men and women [18,27,40].

The 4 health facilities have been selected based on feasibility factors such as patient volume and mobile network coverage, representativeness of diverse service delivery contexts, and requirements of the stepped-wedge design to ensure adequate clusters while minimizing contamination. They serve a total of 1899 men living with HIV aged 18‐55 years: Mbita (n=758), Ogongo (n=294), Ndhiwa (n=598), and Pala (n=249). The average distance between each neighboring facility is 25 km. Mbita and Ndhiwa subcounty hospitals are situated in an urban setup, while Ogongo and Pala subcounty health centers are located in relatively rural setups.

### About Nishauri mHealth Intervention

**Figure 2.**
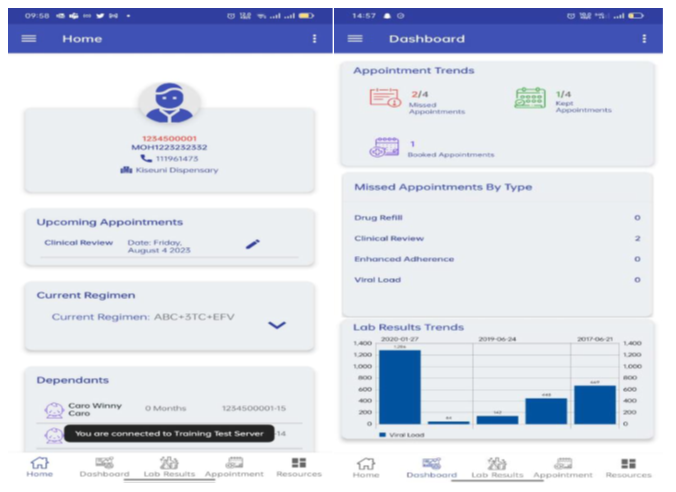
Screenshot of the Nishauri mobile health app showing the home and dashboard interfaces.

The Nishauri mHealth intervention is a client-centered digital platform developed by Palladium, in collaboration with Kenya’s Ministry of Health, and funded by the President’s Emergency Plan for AIDS Relief through the Centers for Disease Control and Prevention, to enhance HIV care and treatment outcomes. Nishauri supports key aspects of HIV care management, including appointment scheduling, ART referrals, and ongoing patient engagement. Its core functionalities include automated appointment and medication adherence reminders. It also enables tailored health education messages, 2-way communication between patients and health care providers, and behavior-change communication strategies to support retention in care. Patients with HIV using Nishauri can access their treatment engagement information and schedule or reschedule appointments through the app. A screenshot of the Nishauri app, captured directly from the live platform, is presented in Figure 2 for illustrative purposes.

Nishauri is the Ministry of Health–endorsed scale-up of Kenya’s earlier Ushauri system, which was piloted to support appointment reminders, treatment adherence messaging, and defaulter tracing through SMS, web, and mobile interfaces [19]. Building on the functionality and adoption of Ushauri, Nishauri is a client-centered mobile app that integrates in real time with the Kenya electronic medical record system and the Ushauri platform to support appointment scheduling, adherence reminders, 2-way communication, and behavior-change messaging for people living with HIV [19,20].

### Conceptual Framework and Theoretical Grounding

**Figure 3.**
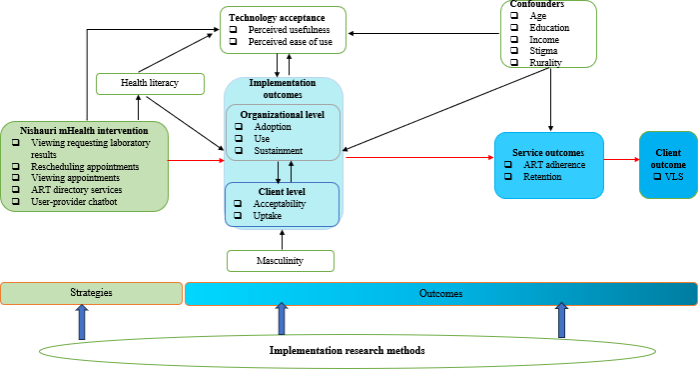
Study conceptual framework. ART: antiretroviral therapy; mHealth: mobile health; VLS: viral load suppression.

This study is guided by an integrated conceptual framework (Figure 3) drawing on Connell’s theory of hegemonic masculinity [29,41] and the TAM. Hegemonic masculinity provides a lens for understanding how dominant gender norms, such as expectations of strength, self-reliance, and economic provision, influence men’s engagement with HIV care and perceptions of digital health interventions. TAM informs the assessment of how perceived usefulness and ease of use of the Nishauri mHealth platform shape adoption and sustained use. These frameworks are integrated to examine how masculine norms modify technology acceptance and implementation outcomes, including adoption, acceptability, and engagement, ultimately influencing HIV care outcomes among men.

### Stepped-Wedge Intervention Approach

#### Strategy

A stepped-wedge design was chosen to allow all facilities to receive the intervention, accommodate phased training and app deployment, minimize contamination between sites, and enable within-facility comparisons over time while controlling for secular trends. The 4 CCCs will undergo a standardized readiness assessment, and phased implementation of the intervention will proceed in sequence according to facility readiness, with sites transitioning from standard care to the mHealth intervention arm over an 8-month period. The phased rollout will occur at 2-month intervals across the 4 participating facilities, resulting in 5 study periods (1 baseline and 4 intervention steps) over approximately 8 months. The 2-month interval was selected to align with routine HIV clinic visit schedules in Kenya [18], allow sufficient time for provider training and participant onboarding, and permit stabilization of app engagement before subsequent facility transitions. This interval is consistent with stepped-wedge evaluations of health service and digital interventions [42] and supports the detection of short-term changes in missed clinic visits and adherence behaviors while accounting for secular trends.

We acknowledge that the 2-month step length is relatively short and may limit detection of longer-term intervention effects within each wedge; however, this interval was selected for programmatic feasibility and alignment with routine HIV care cycles, and outcome measures were therefore chosen to capture proximal, short-term indicators of engagement and retention.

After baseline, all men who access HIV care from each facility will be trained on how to install and use the Nishauri app by adherence counselors or peer educators already trained by Palladium Group. This will happen during clinic education sessions as well as community outreaches organized through community ART (support) groups. Peer educators, through the community support groups, will provide follow-up support to ensure that all eligible men are reached. Contamination will be minimized by staggering rollout, restricting app activation to intervention sites, and monitoring cross-facility clinic attendance. We will track each participant’s primary facility at enrollment and confirm the facility where ART is routinely dispensed. Study inclusion will be restricted to men who are primarily engaged at 1 of the 4 participating facilities during the intervention period. Any participant who routinely receives care at multiple facilities will be carefully documented, and their data will be linked to the facility where they are primarily enrolled. These procedures are consistent with both national treatment guidelines and best practices for stepped-wedge designs in HIV implementation research.

Due to its facility-level implementation, individual-level exclusion criteria will not apply, as specified for the pre- and postassessment, which involves individual-level measurements and participant follow-up.

#### Outcomes

The primary clinical outcome will be missed clinic appointments, while secondary outcomes will include ART adherence (30-day percent visual analog), pharmacy refill consistency (medication possession ratio), and VLS—plasma HIV RNA <200 copies per milliliter. CCC data will be retrieved from facility electronic medical records and patient files, focusing on routinely collected HIV care indicators.

#### Implementation Outcomes

Guided by Proctor’s implementation outcomes framework, this study seeks to evaluate multiple implementation outcomes of the Nishauri mHealth intervention using a mixed methods approach. The primary implementation outcome will be adoption. Adoption will be measured as completion of app installation and account activation within 60 days of introduction. Acceptability (the perceived satisfaction and appropriateness of the intervention among users and providers) and use will be assessed through validated survey instruments, including the System Usability Scale and intervention acceptability measures, alongside qualitative data from FGDs with users and providers. Early indicators of sustainment are assessed through continued engagement metrics over the study period and stakeholder perspectives on long-term integration and scalability, acknowledging that full sustainment extends beyond the study time frame.

#### Stepped-Wedge Analysis

We will analyze intervention effects using intention-to-treat principles and generalized linear mixed models appropriate to each outcome. Models will include a fixed effect for time period (step) to account for secular trends, a fixed indicator for intervention exposure during each step (within-cluster), and random intercepts for clusters (facilities) to account for within-cluster correlation. Where appropriate, we will include random slopes for time if supported by the data. Covariates prespecified for adjustment include patient-level baseline characteristics (age, gender, income, education, and stigma) and facility-level covariates (facility size and urban or rural). We will use robust SEs (cluster-robust SEs) to ensure valid inference with a limited number of clusters. Additional sensitivity analyses will include per-protocol analyses and models testing for time-by-intervention interaction to explore whether effect sizes change across steps. Primary inference will be 2-sided with *α*=.05. Analyses will be performed in Stata (StataCorp LLC; xtmelogit/xtmixed), and code will be archived with the study repository for reproducibility.

#### Power Estimation for Stepped Wedge Analysis

Sample size and power were estimated for the stepped-wedge cluster design using simulation under a Hussey-Hughes mixed effects logistic model. With facility totals of 1899—Mbita (n=758), Ogongo (n=294), Ndhiwa (n=598), and Pala (n=249), assuming baseline missed-appointment proportion (p0) of 20% (0.20) and 5 periods, the study has ~80% power to detect an intervention effect (missed visits) corresponding to an odds ratio of 0.75, assuming intracluster correlation coefficient=0.02 (*α*=.05). Sensitivity analyses indicated that power remained ≥70% for odds ratios of 0.80 and intracluster correlation coefficient values up to 0.05 and exceeded 90% for odds ratios of ≤0.70, supporting the robustness of the proposed sample size.

### Pre- and Postassessment of Implementation Outcomes

#### Participants and Recruitment

The primary study population will comprise men who initiated HIV care and treatment in 2016 or later, following the updated national HIV treatment guidelines. Eligible participants must be between 18 and 55 years of age, own a smartphone or tablet, and be able and willing to provide written informed consent (Multimedia Appendix 1). Men with severe comorbidities or chronic coinfections (eg, cancer or hypertension) that may affect the adoption or use of mHealth interventions will be excluded. Only men who are primarily enrolled at 1 of the 4 participating facilities during the study period will be included. Participant recruitment in each CCC will proceed according to probability proportional to size sampling until the required sample size of 347 participants is reached. Recruitment will occur during routine clinic visits in each facility before the stepped-wedge rollout of Nishauri. Trained study staff or peer educators will introduce the study to clients, provide study information, and collect contact information from those expressing interest. Screening for eligibility will take place in private, either in a clinic or at a location convenient and confidential for the participant. Those who are eligible and interested will then undergo written informed consent.

#### Quantitative Data Collection and Storage

Quantitative data will be collected at baseline, at 8-month follow-up, and via the Nishauri app data. At baseline, potential participants will be screened for eligibility. Eligible participants will provide written informed consent and locator information before undergoing a short baseline survey lasting approximately 40 minutes. The survey includes validated and pilot-tested modules on sociodemographics, technology acceptance, HIV-related stigma, health literacy, masculinity norms, the System Usability Scale, and intervention acceptability. The survey will be administered electronically using tablets with the REDCap (Research Electronic Data Capture) platform, hosted by the San Francisco Coordinating Center. Data will be collected by trained research assistants (RAs), who were locally recruited and had prior experience in health or social science research. RAs have backgrounds in public health or related disciplines; are fluent in English, Kiswahili, and Dholuo; and will receive study-specific training on the study protocol, ethical conduct of research, informed consent, and standardized data collection procedures prior to data collection. RAs will perform daily quality checks on all forms to identify and resolve errors prior to secure upload to REDCap servers. These servers are firewall-protected, regularly backed up, and housed in a secure environment.

Throughout implementation at each site, app-based metrics will be assessed to capture implementation outcomes related to acceptability and uptake of the Nishauri intervention. Facility-based data will be collected, including who gets the intervention, the date of training, and the date of app installation. App analytics will be used to capture patterns of engagement and uptake among participants. This includes the number of log-ins per user, frequency of use over time, cumulative duration of app sessions, and responsiveness to reminders and notifications (whether SMS or in-app prompts were opened or acted upon). Aggregate metrics such as average log-ins per month, median engagement time, and drop-off points will be analyzed to assess acceptability and sustained use across the study period.

All data will be transferred securely to the San Francisco Coordinating Center network via a secure File Transfer Protocol site under a formal data transfer agreement between the University of California, San Francisco, and the local research center. Data will be stored on encrypted, password-protected servers with limited access, using study ID numbers in place of participant names. Physical documents will be stored securely onsite, and all sensitive data will be separated from identifying information to ensure confidentiality.

#### Quantitative Data Analysis

Descriptive statistics will be used to summarize demographic variables, intervention adoption, and predictors of adoption (health literacy, technology acceptance, and masculinity). A participant will be considered to have adopted Nishauri if he has completed device installation and created or activated an account within 60 days of being trained or introduced to the app. Participants who occasionally attend multiple facilities will be documented, and their data will be analyzed based on their primary facility of care. This aligns with national HIV care guidelines, which encourage patients to register at a single facility for longitudinal HIV treatment and monitoring.

#### Power Calculation for the Pre- and Postassessment

With 347 participants, an anticipated 10% attrition rate (effective n≈312), and a primary binary uptake outcome, the study will have adequate power to examine associations between key predictors (health literacy, technology acceptance, and masculinity) and adoption of Nishauri. Assuming an uptake rate of ~70% (adopters: n≈243; nonadopters: n≈104) as seen in other studies [22,23], we will have >80% power (*α*=.05) to detect moderate associations corresponding to odds ratios of roughly 1.7‐2.0 or higher, depending on predictor prevalence. Sensitivity analyses showed that with effective sample sizes ranging from 280 to 330 participants, the study would retain ≥80% power to detect odds ratios between 1.7 and 2.0, depending on predictor prevalence.

#### Retention Strategy

Participants will receive automated appointment reminders via the Nishauri platform and additional study reminders 1 week prior to their scheduled 8-month follow-up visit. For missed visits, trained study staff will attempt to contact participants by phone call or SMS text message to reschedule as soon as possible. If participants cannot be reached remotely, study staff will arrange an in-person follow-up at a safe and confidential location. Participants who relocate outside the study area will be contacted to facilitate follow-up data collection at an appropriate alternative site. These strategies are designed to minimize loss to follow-up and support sustained engagement with both the intervention and routine HIV care.

### Focus Group Discussions

#### Qualitative Data Collection

To complement the quantitative findings, 5 to 6 FGDs with approximately 6‐8 participants per group will be conducted in person by trained and qualified RAs and the principal investigator (DO). The FGDs will comprise 2‐3 with men living with HIV, 2 with health care providers (nurses, adherence counselors, or peer educators), and 1 with app developers. FGD sampling among men living with HIV will be stratified by factors such as age, ART adherence, and proportion of missed clinic visits (Table 1). We will invite 10 participants to each FGD and expect at least 6 to participate in each group. This sample size is expected to achieve thematic saturation in this study context, based on qualitative research standards [43] and was chosen based on guidelines, suggesting that 4‐6 FGDs are sufficient to reach thematic saturation in studies of similar scope and target populations [43-45]. This sampling strategy balances the need for rich qualitative data with feasibility considerations, allowing for in-depth exploration of implementation experiences across stakeholder groups. FGDs will continue until no new themes emerge, ensuring comprehensive coverage of barriers, facilitators, and contextual factors influencing the adoption, use, and sustainment of the Nishauri mHealth intervention.

To preserve the integrity of the data, most FGD participants will be different from those in the quantitative survey arm to reduce contamination, social desirability bias, and respondent fatigue. However, a limited number of individuals who participated in the pre- and poststudy may be included in FGDs to help explain emerging patterns in the data, as this study uses an explanatory mixed methods approach. Overlap will be explicitly tracked, and all analyses will account for repeated participation when interpreting themes, ensuring that qualitative findings complement, rather than double-count, quantitative data. Health care providers and app developers will also be purposively sampled to ensure representation of different implementation perspectives across the system.

The sampling plan and rationale for the FGDs is shown in Table 1. The FGD guides (Multimedia Appendix 2) have been organized thematically and include open-ended questions and follow-up probes to facilitate in-depth exploration of participants’ experiences with the intervention. We will explore contextual factors, including masculinity, stigma, and social norms, that influence the adoption, use, and sustainment of the Nishauri mHealth intervention. The FGDs will be audio-recorded using digital recorders and conducted in private settings to ensure confidentiality and adherence to ethical standards.

#### Qualitative Data Analysis

Audio recordings will be transcribed verbatim and translated into English and analyzed using Dedoose. We will use a thematic analysis approach combining deductive coding guided by Proctor’s implementation outcomes framework [36,37] with inductive coding to capture emergent themes not covered by the framework. Descriptive statistics will summarize FGD participant demographics. Narrative summaries, tables, diagrams, and cross-case comparisons will be used to deepen interpretation. Triangulation with app analytics (eg, proportion of missed visits and use metrics) and member checking will enhance credibility. This study uses an explanatory sequential mixed methods design, using quantitative data to assess intervention uptake and outcomes, followed by qualitative inquiry to explain how factors like masculine norms, stigma, and system-level challenges influence these patterns. Qualitative findings will be integrated with application data from the Nishauri dashboard to uncover underlying mechanisms and contextual factors shaping implementation outcomes. Standard operating procedures will guide FGD facilitation, qualitative data handling, and analysis.

### Dissemination Plan

Study findings will be shared with participants, health care providers, Homa Bay County Ministry of Health officials, and other key stakeholders. Results will be disseminated through presentations at local and international conferences focused on HIV prevention, treatment, and behavioral medicine, as well as through publication in peer-reviewed journals. The study team has established an authorship agreement outlining contributions to primary and secondary publications. Participants’ identities will remain confidential and will not appear in any reports or publications. Direct feedback will also be provided to research participants and community partners to ensure inclusive and transparent communication of the findings.

### Ethical Considerations

This study will be conducted in accordance with the ethical principles of the Belmont Report—respect for persons, beneficence, and justice. Ethics approval was obtained from the Maseno University Scientific Ethical Review Committee (MSU/DRPI/MUSERC/01502/25), which serves as the primary institutional review board. All study materials, including the study protocol, data collection tools, and any protocol amendments, will be submitted to the institutional review board for review and approval prior to implementation. In addition, administrative and institutional approvals were obtained from the National Commission on Science, Technology and Innovation, Homa Bay County—Department of Health, the participating health facilities (Mbita, Ndhiwa, Ogongo, and Pala subcounty hospitals), and the University of California, San Francisco Department of Epidemiology and Biostatistics. All study staff will complete training in human subjects’ protection and Good Clinical Practice prior to study initiation. Written informed consent will be obtained by trained RAs before any study procedures are conducted. Consent forms will be available in English, Dholuo, and Swahili, and participants will choose their preferred language. Literate participants will read and sign the form; those with limited literacy will have the form read aloud in the presence of an impartial witness who will document the consent accordingly. All participants will receive a copy of the signed consent form, or if deemed unsafe to keep, may store it at the clinic and receive a contact slip for future reference. Participants will be reimbursed Ksh 300 (US $2.32) for their time. Strict confidentiality measures will be observed, given the sensitivity of HIV-related data. Personal identifiers will be anonymized or pseudonymized, and all data will be stored in secure, encrypted systems accessible only to authorized, trained personnel. Participants will be informed of confidentiality protections during the consent process. Hard copies of study documents will be stored securely for 5 years before being shredded; electronic and audio files will be permanently deleted at that time.

## Results

The study obtained institutional review board approval on July 28, 2025. Baseline data collection began on September 8, 2025, and has been completed in 3 health facilities and is anticipated to end by January 31, 2026. A total of 307 men living with HIV were recruited across the 4 clinics for the pre- and postquantitative assessment. Follow-up and app use monitoring are ongoing in the first facility. Follow-up data collection is expected to be completed in August 2026. Analysis is projected to be concluded in the third quarter of 2026, and the results will be submitted for publication at the end of 2026. Findings are anticipated to inform gender-responsive adaptations of digital health strategies and guide policy recommendations for scaling mHealth interventions for men living with HIV in Kenya.

## Discussion

### Principal Findings

This study is guided by a conceptual framework integrating Proctor’s implementation outcomes framework, TAM, and the masculinity model (Figure 3). It is designed to generate evidence on the implementation and impact of the Nishauri mHealth intervention on HIV care and treatment among men living with HIV in Homa Bay County, Kenya. With this model guiding data collection and analysis, we anticipate findings that will address critical gaps in understanding how mHealth tools perform in resource-constrained, gendered contexts.

By identifying both individual- and system-level factors that influence the adoption, use, and sustainment of the Nishauri platform, this study will generate valuable insights into the dynamic interplay between client- and organizational-level implementation outcomes. Barriers such as limited digital literacy, stigma, poor network coverage, and workflow integration challenges, as well as facilitators like ease of use, perceived usefulness, provider support, and trust in the health system, will be explored in depth. These findings will inform the development of gender-responsive, culturally grounded adaptations to Nishauri as well as other digital health strategies aimed at improving long-term health outcomes for men living with HIV in Kenya and advancing national efforts to close gender gaps in HIV care.

We hypothesize that there will be measurable improvements in HIV service outcomes, particularly ART adherence and clinic retention, among men exposed to the intervention, compared to control periods within the stepped-wedge design. These outcomes will be interpreted in light of confounding variables, such as age, education, and stigma, and supported by app analytics and chart review data. Furthermore, the client outcome of VLS will offer a critical downstream indicator of the intervention’s potential clinical impact.

This study will contribute critical insights into how masculine norms shape the implementation and impact of digital health tools for HIV care. By unpacking how behavioral constructs such as autonomy, stoicism, and fear of vulnerability influence engagement with mHealth interventions, the findings will inform the development of more gender-responsive and contextually grounded digital strategies. These insights have the potential to guide national policies and programs aiming to close gender gaps in HIV care by tailoring interventions to the unique needs and experiences of men.

By integrating implementation science and gender theory within a mixed methods design, this study will generate a nuanced understanding of individual- and system-level factors influencing the success of mHealth tools among men in HIV care. While the findings may be context-specific to Homa Bay County, they offer valuable insights to inform the design and scale-up of culturally relevant, gender-sensitive digital health strategies in Kenya and similar low-resource settings. Future research should build on these results to codevelop scalable interventions and policy frameworks that support sustainable integration into routine HIV care for men.

### Strengths

This study has several strengths, including its use of a stepped-wedge design within real-world public health settings, enhancing both rigor and relevance. By focusing on men, an underserved population in HIV care, and guided by Proctor’s implementation outcomes framework, the study offers a structured analysis of adoption, use, and sustainability. The explanatory sequential mixed methods approach, combined with mHealth app data, allows for deep insights into both outcomes and underlying mechanisms. Additionally, the study’s gender-informed lens and alignment with national HIV priorities position it to generate policy-relevant evidence for more effective and equitable digital health interventions.

### Limitations

While this study uses a stepped-wedge cluster randomized design, several limitations should be noted. First, the number of clusters (health facilities) is small, which may limit statistical power and the ability to control for facility-level confounding. The small number of clusters limits the precision with which facility-level confounders can be modeled and reduces degrees of freedom for estimating random effects. As a result, adjustment for cluster-level confounding is restricted to a limited set of prespecified facility characteristics (eg, size and urban-rural setting), and residual confounding at the facility level cannot be entirely ruled out. To mitigate this limitation, analyses will include fixed effects for time, intention-to-treat estimation, and triangulation with qualitative data to support interpretation of observed effects. Second, reliance on self-reported survey measures for some implementation outcomes (eg, acceptability and adherence) and masculinity factors may introduce recall or social desirability bias. Third, inclusion criteria requiring smartphone or tablet ownership may exclude more men from resource-limited countries, potentially limiting the generalizability of findings. Finally, variations in how the intervention is implemented across facilities—due to staffing, infrastructure, or engagement differences—may influence observed effects. Despite these limitations, the study’s mixed methods approach and triangulation of data sources will enhance the robustness and contextual relevance of the findings.

### Conclusions

The Nishauri mHealth intervention was developed to address persistent gaps in HIV care engagement and outcomes among people living with HIV in Kenya, a group historically underserved by conventional HIV programming. While mHealth solutions have shown promise in improving HIV-related outcomes, few have been systematically implemented or evaluated with a gender lens in high-burden settings like Homa Bay County. This study will evaluate the real-world implementation and effects of Nishauri using a mixed methods, stepped-wedge design—generating evidence on both process and clinical outcomes while integrating perspectives from users, providers, and developers. If successful, Nishauri could offer a scalable, gender-responsive mHealth model that enhances retention, adherence, and viral suppression among men. The findings will inform future efforts to strengthen digital health strategies and promote more equitable HIV care for men in Kenya and similar resource-limited settings.

## Supplementary material

10.2196/85279 Multimedia Appendix 1 Informed consent form.

10.2196/85279 Multimedia Appendix 2 Focus group discussion guide.

10.2196/85279 Checklist 1 SPIRIT checklist.

## Abbreviations

ART: antiretroviral therapy
CCC: Comprehensive Care Clinic
FGD: focus group discussion
mHealth: mobile health
RA: research assistant
REDCap: Research Electronic Data Capture
SPIRIT: Standard Protocol Items: Recommendations for Interventional Trials
TAM: technology acceptance model
VLS: viral load suppression
