# Supplementary material for: Assessing the Implementation and Potential Effects of the Nishauri mHealth Intervention on HIV Care Among Men in Homa Bay County, Kenya: Protocol for a Mixed Methods Study
Source: JMIR Res Protoc. 2026 Mar 24;15:e85279. doi: 10.2196/85279 (PMC13012606; doi:10.2196/85279)
Supplement: Multimedia Appendix 1 [file resprot-v15-e85279-s001.pdf]

Survey ICF_ Quantitative Surveys_ ENGLISH

Title of the Study: *Assessing the Implementation and Effects of Nishauri m-Health Intervention on HIV Care and Treatment Outcomes Among Men living with HIV in Homa Bay County, Kenya*

Purpose of the study
We are from Maseno University and work in collaboration with University of California San Francisco, in the United States of America. We are conducting a research study to assess the implementation of Nishauri, a m-Health intervention providing HIV care support via mobile technology, and its effects on clinical outcomes, including retention, adherence, and viral load suppression among men living with HIV in Homa Bay County.
You are being invited to participate in this study to help us understand the implementation and effects of Nishauri m-Health intervention among men living with HIV like you.

What do I have to do if I agree to participate?
If you agree to take part, you will be asked to participate in a survey today regarding your experiences with HIV care and m-Health in general which will take about 40 minutes. A few weeks later, a peer educator will support you to install and begin using the Nishauri app. If you install the app, you will engage with it, which may include receiving and rescheduling appointments and treatment support through your mobile phone. We will ask you to allow us to review your records at the health facility and in the app dashboard (with personal identifiers removed) every month to assess your HIV treatment history and outcomes and your engagement with the app. After 6 months, you will participate in a final survey on your perceptions of and experiences with Nishauri or m-Health in general. Your responses and participation will help us assess the implementation outcomes and effects of the intervention.

What are the potential risks?
Some of the questions during the survey or in relation to the intervention may make you feel uncomfortable. You do not have to answer any questions that make you uncomfortable.
We have implemented procedures to ensure your confidentiality is maintained. However, there is a very small chance that confidentiality could be compromised. All study staff are trained in participant confidentiality, and your personal information will not be linked to your responses. Additional protective measures are outlined below.

What are the potential benefits?
You may not receive any direct personal benefit from participating. However, the information gathered in this study will help improve m-Health interventions and HIV care in future for men.

What about confidentiality?
This study has been approved by the Maseno University Ethical Review Committee (MUERC) and National Commission on Science and Technology (NACOSTI). If you agree to participate, all information collected will be kept strictly confidential. Your name will not appear on any data collection forms or records. Access to your data will be restricted to authorized study staff, who are trained to protect your confidentiality. Study records may be reviewed by authorized personnel from the Ethics Review Boards and other regulatory bodies but no personal identifiers will be disclosed.

Will I be given anything for taking part?
At the end of the study, you will receive Ksh 300 for your time taken to answer the questions.

Are there any costs?
There is no cost for participating in this study.

Can I leave the study?
Your participation is completely voluntary. You are free to withdraw from the study at any time without penalty or loss of benefits. If you choose not to participate or decide to withdraw, it will not affect your access to HIV care or any other health services.

Questions/Points of contact
If you have any questions, please feel free to ask. We are happy to provide clarification.
For more information or if you have any concerns about the study, please contact Dan Omollo at 0724-734312 or danodomany@gmail.com
If you have questions about your rights as a participant, you may contact Maseno University Ethical Review Committee at + 254 57 351 622, EXT. 3050, Email: muerc-secretariate@maseno.ac.ke

CONSENT STATEMENT
I have read this form, or it has been read to me, and I have had the opportunity to discuss it with the study team. I have had my questions answered and have received a copy of this consent form. By signing below, I agree to participate in the study.
I understand that my participation is voluntary, and I may withdraw at any time without affecting my access to services.

______________	______________________________	______________
Date	Name of Participant	Signature or Thumb Print
______________	______________________________	______________
*Date	*Name of Witness	*Signature
______________	______________________________	______________
Date	Name of Person Obtaining Consent	Signature

* Witness is only required if participant is illiterate
