# Supplementary material for: Assessing the Implementation and Potential Effects of the Nishauri mHealth Intervention on HIV Care Among Men in Homa Bay County, Kenya: Protocol for a Mixed Methods Study
Source: JMIR Res Protoc. 2026 Mar 24;15:e85279. doi: 10.2196/85279 (PMC13012606; doi:10.2196/85279)
Supplement: Multimedia Appendix 2 [file resprot-v15-e85279-s002.pdf]

Focus Group Discussion (FGD) Guide_ ENGLISH

OPENING *(MODERATOR)*

- Welcome participants and thank them for joining.
- Ensure informed consent has been obtained from each participant.
- Assign cards with numbers, letters or pseudonym to each participant to anonymize the discussion responses.

Begin the audio-recording.

State the FGD type (group being interviewed), venue, start time and date.

Read:

Good [*morning/afternoon*], and thank you for joining us. My name is [*moderator's name*] and my colleague is called [*note-taker's name*]. I'll be guiding our discussion today. We're here to talk about the Nishauri app and its role in supporting HIV treatment for men in Homabay County. This discussion is part of a research project aiming to understand what helps or hinders people in using Nishauri, so we can improve the app and how it's rolled out in the future. Your experiences and views are very important to us. There are no right or wrong answers, and everything you share will be kept confidential. We ask that you speak freely, one person at a time, and respect each other's views. With your permission, we'll record this discussion to help us remember your insights accurately, but the recordings will be kept secure and private. Participation is completely voluntary, and you can skip any question or stop at any time. Before we start, does anyone have any questions?

<u>GROUP 1: Men Living with HIV who used Nishauri (ADOPTERS)</u>

1. General Experience

- *Main question*: How did you first hear about the Nishauri app?
    - *Probes:*
        - Was it from a health worker, another client, community health volunteer?
        - What was your first reaction?
    - *Follow-up:* What made you decide to install it (or not) right away?

- *Main question*: Can you describe how you used the app?
    - *Probes:*
        - Which features did you use most (e.g., reminders, messages, lab results)?
        - Did you use it daily, weekly, only when needed?
    - *Follow-up:* What was most helpful or least helpful?

2. Facilitators to Adoption and Use

- *Main question*: What encouraged or motivated you to start using Nishauri?
    - *Probes:* Was it curiosity, health concerns, provider encouragement? Did it feel private and safe to use?
    - *Follow-up:* Did any male peers or support groups influence your decision?

- *Main question*: Was there anything about the app that made it easy to use?
    - *Probes:* Simple design? Clear messages? App size? Accessibility with your phone or network?
    - *Follow-up:* Would you say it was easier or harder than other apps you've used?

- *Main question*: Did anyone support or encourage your use of the app (e.g., peers, health workers)?

    - *Probe:* Can you tell me more about how they encouraged you?
      (For example, did they explain how to use the app, help you install it, or share their own positive experience?)
    - *Follow-up:* How did their support influence your decision to start—or keep—using Nishauri? (Did it make you feel more confident, motivated, or reassured about using app?)

3. Barriers to Continued Use

- *Main question*: Were there times you stopped using the app? Why?
    - *Probes:*
        - Lost your phone? Data costs? App bugs?
        - Felt discouraged, overwhelmed, or demotivated?
    - *Follow-up:* How long did you stop, and what brought you back?

- *Main question*: Did you face any concerns using the app?
    - *Probes:*
        - Worried about someone seeing your HIV-related messages?
        - Fear of stigma from family/friends who might access your phone?
    - *Follow-up:* What would make you feel more secure or confident using it?

4. Sustainability and Effects

- *Main question*: Are you still using Nishauri today? Why or why not?
    - *Probes:*
        - What makes you keep using it?
        - If not using: what changed? Was it personal, technical, or health-related?
    - *Follow-up:* Would you recommend it to another man living with HIV?

- *Main question*: What effects/changes has the app had on your HIV care?
    - *Probe:* Did it help with medication reminders, clinic attendance, communication with providers?
    - *Follow-up*: How does it compare to before you had the app?

5. Recommendations

- What features or changes would make the app more useful for men like you?
    - *Probe:* Any parts you found confusing or missing?
    - *Follow-up:* How would those changes help you or others use it better?

- How can health facilities better support men in using such apps?
    - *Probe:* Did anyone at the clinic ever help you with the app?
    - *Follow-up:* What kind of support would have made it easier to use?

GROUP 2: Men Living with HIV who never used Nishauri (NON-ASOPTESRS)

1. Awareness and First Impressions

- *Main question*: Have you heard of the Nishauri app? What did you think about it?
  - *Probes:*
    - Where did you hear about it (clinic, peer, radio)?
    - Did it sound helpful or unnecessary?
  - *Follow-up:* Did anyone ever encourage or pressure you to use it?

2. Barriers to Adoption

- *Main question*: What stopped you from trying the app?
  - *Probes:*
    - No smartphone? Not comfortable with apps? Didn't see the need?
    - Distrust of apps with HIV info?
  - *Follow-up:* If you had the right phone or support, would you reconsider?

- *Main question*: Did anything worry you about using Nishauri?
  - *Probes:*
    - Fear of being "outed" by notifications or app content?
    - App taking up space, draining battery, or using too much data?
  - *Follow-up:* Have you ever used other health-related apps?
- Were there concerns about confidentiality, phone access, or relevance?
  - *Probe:* Any worry that someone else might see your messages or data?
  - *Follow-up:* How did these concerns affect your use or decision to use the app?

- Did anyone talk to you about the app (health worker, friend)? What was said?
  - *Probe:* Did they encourage or discourage you from using it?
  - *Follow-up:* Did what they said influence your decision in any way?

3. Trust and Engagement

- *Main question*: How do you feel about using mobile phones for health services?
  - *Probes:*
    - Do you prefer face-to-face advice?
    - Do you trust information sent through apps?
  - *Follow-up:* What would help build your trust or make you comfortable while using an app like Nishauri?

4. Suggestions for Improvement

- *Main question*: What would make you or other men more willing to try such an app?
  - *Probes:*
    - Easier instructions, simpler language?
    - More assurances of confidentiality?
  - *Follow-up:* Would you be open to a demo or peer-led introduction? How can the app be introduced in a better way?

GROUP 3: Health Care Providers (HCP)

1. Experience and Role
- *Main question*: How were you involved in Nishauri's rollout?
    - *Probe:* Did you train clients, troubleshoot issues, give feedback to developers?
    - *Follow-up:* Did your workload increase or decrease because of the app?

2. Facilitators to Implementation
- *Main question*: What helped you and your team successfully introduce the app?
    - *Probe:* Strong training? Management support? Patient interest?
    - *Follow-up:* Did the men respond positively at first? Any support integrating it in routine care?

3. Barriers to Implementation and Sustainment
- *Main question*: What difficulties did you face in getting men to use the app?
    - *Probe:* Tech literacy? Trust issues? Phone compatibility?
    - *Follow-up:* How did you try to overcome these?

- *Main question*: Are there system-level or resource challenges you've noticed?
    - *Probe:* Issues with staffing, inconsistent internet, app maintenance? Training gaps, staff turnover?
    - *Follow-up:* Were there differences across facilities? What about technical issues (e.g., app bugs, syncing problems)?

4. Impact and Perceived Value
- *Main question*: How has Nishauri influenced patient outcomes or engagement?
    - *Probe:* Improved appointment keeping? Better ART adherence?
    - *Follow-up:* Have you noticed any gender differences in uptake?

5. Sustainability and Recommendations
- *Main question*: What would help sustain or improve the app?
    - *Probe:* Incentives for patients? Training refreshers for staff? More user control over messages?
    - *Follow-ups:*
        - Is the app still being promoted/used in your facility? Why or why not?
        - How can HCPs be better supported in this effort?

GROUP 4: App Developers

1. Design and Development
   - *Main question*: What was the rationale behind creating Nishauri?
     - o *Probe:* Did you conduct user testing or engage male PLHIV during the process?
     - o *Follow-up:* Were there any design decisions influenced by gender-specific feedback?

2. Rollout and Engagement Strategies
   - *Main question*: What strategies did you use to drive/encourage adoption?
     - o *Probe:* Peer champions? In-facility demonstrations? SMS campaigns?
     - o *Follow-up:* What worked best and what didn't?
   - *Main question:* What kind of feedback mechanisms were in place during rollout?
     - o *Probe:* Did you set up any channels for users or health workers to report issues or suggestions?
     - o *Follow-up:* How did your team respond to the feedback—were any updates or changes made based on it?

3. Challenges Faced
   - *Main question*: What were some key implementation hurdles?
     - o *Probe:* Phone types, app updates, literacy, male engagement?
     - o *Follow-up:* Were there any unexpected challenges related to masculinity, privacy, or stigma? Did you adjust the app after feedback?

4. Monitoring, Evaluation and Learning
   - *Main question*: How did you track how well the app was working?
     - o *Probe:* Usage analytics? Qualitative feedback? Support tickets?
     - o *Follow-up:* What user behaviour, metrics or patterns surprised you?

5. Future Plans and Sustainability
   - *Main question*: What is the plan for the future of Nishauri? What do you see as necessary to scale or sustain Nishauri in places like Homa bay?
     - o *Probe:* Integration with EMRs? Local ownership? User customization?
     - o *Follow-up:* Have you engaged policy-makers or funders in this?

Closing Questions for All Groups
- What is one thing you would change about Nishauri?
- What is one reason you'd recommend (or not recommend) it to someone else?
- Any final thoughts you'd like to share?

CLOSING
MODERATOR: State the Ending Time and Read-
"Thank you all for your time and for sharing your honest thoughts and experiences. Your feedback is incredibly valuable and will help us improve how the Nishauri app and similar tools are used to support HIV care. If you have anything else you'd like to add, feel free to speak to us privately afterward. We really appreciate your contribution."
